# Factors influencing healthcare use among poor older females under the Livelihood Empowerment Against Poverty programme in Atwima Nwabiagya District, Ghana

**DOI:** 10.1186/s13104-019-4355-4

**Published:** 2019-06-07

**Authors:** Williams Agyemang-Duah, Justice Kufour Owusu-Ansah, Charles Peprah

**Affiliations:** Department of Planning, Kwame Nkrumah University of Science and Technology, Kumasi, Ghana

**Keywords:** Healthcare utilisation, Poor older females, LEAP programme, Ghana

## Abstract

**Objective:**

While studies show that females utilise more healthcare services in later life, data on their healthcare use predictions are limited in Ghana. This study therefore fills this gap by examining the predictors of healthcare use among poor older females under the Livelihood Empowerment Against Poverty (LEAP) programme in Atwima Nwabiagya District of Ghana. A sample of 156 poor older females was extracted from an Ageing, Health, Lifestyle and Health Services Survey which was conducted between 1 and 20 June 2018 in Atwima Nwabiagya District. Sequential logistic regression models were used to analyse the data.

**Results:**

The fully adjusted model showed that respondents aged 85–89 years (AOR = 0.007, CI 0.001–0.958), those without past illness records (AOR = 0.027, CI 0.002–0.346) and not diagnosed of chronic non-communicable diseases (AOR = 0.003, CI 0.001–0.313) were significantly less likely to utilise a health facility compared with their respective counterparts. Non-vegetables consumers (AOR = 1.2, CI 0.23–2.45) were found to be more likely to utilise healthcare services. These findings have implications for policies towards healthcare use among poor older females in developing countries including Ghana.

## Introduction

As in other countries, older populations are rapidly increasing in Ghana [1–7]. This increase is attributed to prolonged fertility reduction, gradual reduction in mortality and the rise in life expectancy [1, 4, 5]. Coupled with poor healthcare system for older people in developing countries, they are faced with health challenges including chronic diseases, decline in physical functioning which may call for increased utilisation of healthcare services [7].

Although women live longer [8], they tend to report greater morbidity and disability than men [2, 9]. Females’ exposure to diseases and their response to healing interventions are mostly different from those of males because women have a distinctive physiology [10, 11]. Thus, the odds of healthcare utilisation between males and females may differ due to differences in their morbidity conditions [9, 12]. To this end, the design of a gender-based healthcare planning for vulnerable older people has become an important public health policy in low- and middle-income countries [LMICs]. Because women report higher use of healthcare in later life [13, 14], a better understanding of factors explaining their healthcare use could contribute partly to the realisation of the United Nations-health related Sustainable Development Goals. However, studies explaining healthcare use among poor older females are limited in Ghana. This study therefore addresses this knowledge gap by analysing predictors of healthcare utilisation among poor older females aged 65 years or above who are a beneficiary of cash transfer policy under the LEAP programme in Atwima Nwabiagya District of Ghana. The LEAP is a financial transfer policy for households and persons in Ghana that are extremely poor in order to reduce their vulnerabilities [15–18].

Considering the Andersen’s Behavioural Model which comprises predisposing, enabling and needs variables [19, 20], recent Ghanaian studies have grouped factors influencing healthcare use among older people into demographic, socio-economic [21, 22], health-related [7] and health-behaviour factors [13]. Therefore, this study analyses how these four variables explain healthcare utilisation among poor older females in Ghana. These variables and their conceptualisations are explained in this study under the methods section.

## Main text

### Methods

#### Data and sample

Cross-sectional data were extracted from an Ageing, Health, Lifestyle and Health Services (AHLHS) survey conducted between 1 and 20 June 2018 among poor older people aged 65 or above under the LEAP programme in Atwima Nwabiagya District. The AHLHS survey involved 200 respondents in 16 selected communities conducted through cluster and simple random sampling techniques. The 200 respondents were estimated using Miller and Brewer’s [23] formula for sample size calculation. ${\text{n}} = \frac{\text{N}}{{1 + {\text{N}}\left( \propto \right)^{2} }}$, n (sample size), N (sample frame) and ∝ is the margin of error (0.05). Mathematically as follows:

$$\frac{401}{{1 + 401\left( {0.05} \right)^{2} }}$$

$$\frac{401}{2.0025} = 200.25, {\text{approximately }}200.$$

Out of the 200 respondents, 156 were females. The high number of females involved in the AHLHS survey is attributed to the gendered nature of poverty and inequality in Ghana [17]. About 75% of the LEAP beneficiaries in Ghana particularly, in the study area were females [15]. To ensure diverse demographic and socioeconomic characteristics of poor older females under the LEAP programme, all geographical areas within the district were fully represented. Thus, the study area was clustered into three geographical areas (that is north–south–central divide) then simple random sampling was used to select the respondents. A detailed description of the sampling procedure has been reported elsewhere [13].

Questionnaires were developed in English. These questionnaires were subsequently translated into Twi (local language of the respondents) [7], following the World Health Organization guidelines for assessment of instruments [24] which is consistent with previous studies [21, 22]. The translation was performed by the first author and was followed by independent checks and re-checked by the authors to ensure quality control. The questionnaires included demographic, socio-economic, health-related, health behaviour characteristics of the respondents as well as use of formal healthcare over the last one year preceding the survey. The validity of the questionnaire was determined by comprehensively reviewing related literature for language clarity, simplicity and consulting experts in the field of healthcare utilisation. The questionnaire administration was done by three trained research assistants; however the first author monitored the data collection process. The estimated burden time for each questionnaire was between 30 and 40 min. Both written and verbal informed consents were sought from the participants before data were collected.

#### Measures

##### Outcome variable

Formal healthcare utilisation was measured as dichotomous variable indicating ‘no utilisation’ or ‘utilisation’ of healthcare over the past 1 year preceding the survey. Formal healthcare utilisation was defined as seeking medical treatment from a health professional at a facility such as hospitals, clinics, or health centres.

##### Predictor variables

The predictor variables were measured in four areas: demographic, socio-economic, health-related and health behaviour variables. The demographic variables were marital status (1 = single, = 2 married), ethnicity (1 = Akan, 2 = Northerner), age (years) (1 = 65–69, 2 = 70–74, 3 = 75–79, 4 = 80–84, 5 = 85–89, 6 = 90 or above) and religious group (1 = Christianity, 2 = Islam, 3 = African Traditional religion). The socio-economic variables were education (1 = no formal education, 2 = basic school education, 3 = high school education), income (GH¢) (1 = 100 or less, 2 = 101–200, 3 = 201–300, 4 = above 300), enrollment in the National Health Insurance Scheme (NHIS) (1 = Yes, 0 = No) and family support (1 = Yes, 0 = No). Health-related variables were past illness records (1 = Yes, 0 = No), disability status (1 = Yes, 0 = No), chronic non-communicable diseases (NCDs) (1 = Yes, 0 = No) and self-related health (1 = Good health status, 2 = Poor health status). Engagement in physical activity (1 = Yes, 0 = No), consumption of alcohol (1 = Yes, 0 = No), consumption of tobacco (1 = Yes, 0 = No), fruit intake (1 = Yes, 0 = No) and vegetable consumption (1 = Yes, 0 = No) were health behaviour variables. Marital status, enrollment in NHIS, ethnicity, past illness records, alcohol intake and family support were dichotomised on the basis of Gyasi et al. [25]. Consumption of tobacco, fruit intake, chronic NCDs, engagement in physical activity and vegetable consumption were dichotomised following Gyasi [26]. Self-rated health was dichotomised following Subramanian et al. [27], Fonta et al. [28] and Bourne [29]. A comprehensive explanation of the evaluation procedure of the dichotomous variables has been reported elsewhere [25–29].

#### Analytical framework

The data were entered into database and analysed statistically using SPSS software (version 16.0). Descriptive analyses were used to describe the background characteristics of the study sample. Furthermore, logistic regression models were employed to estimate the variables that were associated with healthcare use. Four different sets of models were developed to estimate the predictors of healthcare use. Model 1 consisted of demographic variables. Model 2 comprised socio-economic variables plus all variables in Model 1. Model 3 consisted of all variables in Model 2 plus health-related variables. Model 4 (full Model) comprised all variables in Model 3 plus health behaviour variables. Odds ratios (ORs) with 95% CI were reported at a significant level of 0.05 or less.

### Results

#### Socio-demographic characteristics of the respondents

Table 1 presents the socio-demographic characteristics of the respondents. Approximately 30% of the respondents in the district were aged between 65 and 69 years. The majority of the respondents were of Akan ethnicity (84%), single (80.1%), Christians (83.3%), with no formal education (66.7%) and 96.2% had enrolled in the NHIS. About 35.9% received a monthly income of GH¢100 ($20.96)^1^ or less.

**Table 1:** Socio-demographic characteristics of the respondents

Variable	Category	N = 156	%
Age (years)	65–69	47	30.1
	70–74	33	21.2
	75–79	20	12.8
	80–84	22	14.1
	85–89	5	3.2
	90 and above	29	18.6
Ethnic group	Akan	131	84.0
	Northerner	25	16.0
Religion	Christianity	130	83.3
	Islam	23	14.7
	African traditional religion	3	1.9
Marital status	Single	125	80.1
	Marital status	31	19.9
Education	No formal education	104	66.7
	Basic school education	37	23.7
	High school education	15	9.6
Monthly income (GH¢)	100.00 or less	56	35.9
	101.00–200.00	60	38.5
	201.00–300.00	29	18.6
	Above 300	11	7.1
Have you ever registered for health insurance? (NHIS)	Yes	150	96.2
	No	6	3.8

Respondents who were widowed/widower, divorce or separated were considered as single

#### Predictors of healthcare use among the respondents

Table 2 presents results on factors influencing healthcare use among poor older females. In Model 1, the study revealed that traditionalists who may be attached to traditional medicine were significantly less likely to utilise formal healthcare compared with their respective counterparts (AOR = 0.068; CI 0.005–0.879). In Model 2, respondents with no family support in terms of money were significantly less likely to utilise healthcare (AOR = 0.127; CI 0.021–0.780). The introduction of socio-economic variables in Model 2 particularly, family support rendered the association between religion and healthcare use insignificant. This outcome suggests that family support is a good predictor of healthcare use than religion.

**Table 2:** Sequential logistic regression results on the determinants of healthcare use among poor older females

Variable	Model 1		Model 2		Model 3		Full model	
	AOR	95% CI	AOR	95% CI	AOR	95% CI	AOR	95% CI
Demographic								
Age (years)								
70–74^a^	0.845	(0.167–4.276)	0.549	(0.089–3.380)	0.376	(0.049 –2.909)	0.094	(0.003–2.659)
75–79	0.369	(0.073–1.854)	0.260	(0.042–1.632)	0.152	(0.020–1.180)	0.089	(0.004–2.271)
80–84	0.675	(0.121–3.777)	0.271	(0.036–2.035)	0.318	(0.033–3.025)	0.175	(0.004–6.995)
85–89	0.258	(0.021–3.162)	0.227	(0.014–3.625)	0.310	(0.014–2.921)	0.007*	*(0.001–0.958)*
90 and above	0.624	(0.129–3.013)	0.072	(0.072–2.809)	0.351	(0.042–2.921)	0.167	(0.004–7.584)
Ethnicity								
Akan^b^	0.949	(0.229–3.932)	1.325	(0.251–7.001)	1.187	(1.187–7.523)	1.835	(0.069–48.482)
Religious group								
Muslim^c^	0.279	(0.081–0.963)	0.150	(0.060–0.762)	0.324	(0.072–1.420)	0.376	(0.037–3.849)
Traditional	0.068*	*(0.005–0.879)*	0.700	(0.215–1.270)	0.199	(0.004–9.033)	0.005	(0.000–3.855)
Marital status								
Married^d^	2.338	(0.477–11.456)	2.778	(0.527–14.640)	2.713	(0.475–15.506)	3.850	(0.257–57.687)
Socio-economic								
Education								
Basic school^e^			0.938	(0.231–3.807)	0.612	(0.133–2.811)	0.105	(0.009–1.214)
High school			1.906	(0.191–19.031)	0.861	(0.069–10.754)	1.101	(0.011–114.042)
Income (GHS)								
101–200^f^			1.125	(0.270–4.683)	0.873	(0.193–3.940)	0.432	(0.049–3.805)
201–300			0.662	(0.132–3.310)	0.760	(0.118–4.901)	3.730	(0.152–91.581)
Above 300			0.762	(0.095–6.128)	0.667	(0.067–6.664)	0.453	(0.019–10.760)
Uninsured			0.146	(0.017–1.230)	0.246	(0.023–2.657)	0.265	(0.004–16.614)
No family support			0.127*	*(0.021–0.780)*	0.100*	*(0.011–0.918)*	0.029	(0.000–0.313)
Health related								
No past illness (3 months)					0.128*	*(0.028–0.587)*	0.027*	*(0.002–0.346)*
Poor health status					0.507	(0.130–1.969)	0.254	(0.023–2.771)
No NCDs					0.244	(0.051–1.166)	0.003*	*(0.001–0.313)*
No disability					2.293	(0.260–20.224)	14.492	(0.417–503.645)
Health behaviour								
Non-physical activity (1 month)							0.636	(0.064–6.270)
Non-alcohol users (1 year)							0.023	(0.002–1.419)
Non-tobacco smokers (1 year)							94.950	(294–3061. 87)
Non-fruit intakes (1 month)							2.106	(0.097–45.911)
Non-vegetable intake (1 month)							1.2*	*(0.230–2.45)*
Model fitting information								
− 2Log Likelihood	104.403			92.045	80.018		47.924	
Hosmer–Lemeshow χ^2^ (significance)	2.820 (0.901)			3.414 (0.268)	0.390 (0.557)		4.867 (0.772)	
Nagelkerke R^2^	0.132			0.268	6.812		0.672	

Italic values indicate significance of *p* value (*p* < 0.05)

CI, confidence interval; OR, odd ratio; AOR, adjusted odd ratio

* *p* < 0.05

^a^65–69 years is the reference category for age variable

^b^Northerner is the reference category for ethnicity variable

^c^Christianity is the reference category for religious variable

^d^Single is the reference category for marital status variable

^e^No formal education is the reference category for education variable

^f^100 Ghana cedis or less is the reference category for income variable

Model 1 = Demographic variables; Model 2 = All variables in Model 1 plus socio-economic variables; Model 3 = All variables in Model 2 plus health-related variables; Model 4 = All variables in Model 3 plus health behaviour variables

In Model 3, the study found that respondents who received no family support (AOR = 0.100; CI 0.011–0.918) and with no past illnesses (AOR = 0.128, CI 0.028–0.587) were significantly less likely to utilise healthcare. This shows that despite the introduction of health-related factors in Model 3, family support still explained healthcare use among the respondents. Finally, with Model 4, respondents aged 85–89 years (AOR = 0.007, CI 0.001–0.958), with no past illness records (AOR = 0.027, CI 0.002–0.346) and not diagnosed of chronic NCDs (AOR = 0.003, CI 0.001–0.313) were significantly less likely to utilise a health facility compared with their respective counterparts. Non-vegetables consumers (AOR = 1.2, CI 0.23–2.45) were more likely to utilise healthcare services. While the introduction of health behaviour variables in the final model rendered the association between family support and healthcare use insignificant, the association between chronic NCDs and healthcare use was rendered significant. Even though, there was no association between age and healthcare use in Model 3, the final Model observed association between age and healthcare use.

### Discussion

Despite an association between age and healthcare use in this study which is consistent with previous studies [30–33], the finding that respondents aged between 85 and 89 years were significantly less likely to use healthcare was counter-intuitive and appeared inconsistent with our expectation and earlier studies in LMICs. This is because, a study in 16 European countries revealed that the propensity of having multimorbidities increased considerably with advancing age [34]. In general, growing older has implications for health status and older age correlates with rising health challenges [2]. One possible reason for our finding could be the exclusion of poor older men from this study because their inclusion could have yielded a different result. Whereas our finding is relatively new and adds to knowledge, it further draws the attention of researchers to consider the views of both older men and women regarding their healthcare use predictions in Ghana.

The study revealed that respondents with no past illness records were less likely to utilise healthcare services. Poor older females with past illness records may perceive the likelihood of later developing the risks of a disease and may therefore attempt to seek for healthcare. Andersen [20] argued that individuals who experienced varied past health problems have higher odds of using healthcare. Similarly, illness level is noted as the most immediate factor that predicts healthcare use [19, 20, 35]. Consistent with previous studies in developing and developed economies, this study found a significant association between increasing number of chronic diseases and utilisation of healthcare [36–40]. We further found that respondents with no chronic NCDs were less likely to use healthcare [36, 37]. Persons who perceive themselves of being susceptible to a disease are likely to take action, and that individuals may seek intervention for a health problem based on its medical consequences [41]. The study also found that those who did not regularly consume vegetables in the past one month were more likely to use healthcare. This is not surprising as vegetables consumption is generally perceived as having health benefits [42–44]. The findings therefore have implications for policies towards healthcare use among poor older females in developing countries including Ghana.

### Conclusion

This study found that age, past illness, chronic NCDs and vegetable consumption were significantly associated with healthcare use among poor older females. These findings are key to contributing partly to the realisation of United Nations health-related Sustainable Development Goals. We recommended that factors such as age, past illness records, chronic NCDs and vegetable consumption should be considered in policy formulation on healthcare of poor older females in Ghana.

## Limitations

Data were collected through self-reporting which may lead to potential recall and reporting biases. To minimise the limitation, we performed validation checks by comparing the original questionnaires several times with the data entered in the SPSS software to ensure quality control. Due to the cross sectional nature of the study, the direction of the causal relationship between healthcare use and proximate factors could not be determined. Also, poor older men were excluded from this study. Thus, future studies should examine gender gap in healthcare use among poor older people under the LEAP programme in Ghana.

## Data Availability

The datasets used and/or analyzed during the current study are available from the corresponding author on reasonable request.

## Abbreviations

AHLHS: Ageing, Health, Lifestyle and Health Services
LEAP: Livelihood Empowerment Against Poverty
NCDs: Non-Communicable Diseases
NHIS: National Health Insurance Scheme

## Footnotes

1 Exchange rate as at the time of the field survey (GH¢ 4.77 = US$1.00).
